# Supplementary material for: Prediction of hospital readmission of multimorbid patients using machine learning models
Source: PLoS One. 2022 Dec 22;17(12):e0279433. doi: 10.1371/journal.pone.0279433 (PMC9779015; doi:10.1371/journal.pone.0279433)
Supplement: Attachment — Submitted filename: Response to reviewers.pdf [file pone.0279433.s002.pdf]

Jules Le Lay
École des Mines de Saint-Étienne
158 cours Fauriel,
42100 Saint-Étienne

Dear Editors of the PLOS ONE journal,

Thank you once again for giving us the opportunity to submit a revised version of our manuscript, titled “Prediction of Hospital Readmission of Multimorbid Patients Using Machine Learning Models” to your journal. This article was written by Le Lay, Alfonso-Lizarazo, Augusto, Bongue, Masmoudi, Xie, Gramont, and Célarier. We would like to thank you and the reviewers for taking the time to review and comment this paper.

You will find here after our responses to your comments and to the reviewers comments.

Editor’s comments:

Please review your reference list to ensure that it is complete and correct. If you have cited papers that have been retracted, please include the rationale for doing so in the manuscript text, or remove these references and replace them with relevant current references. Any changes to the reference list should be mentioned in the rebuttal letter that accompanies your revised manuscript. If you need to cite a retracted article, indicate the article’s retracted status in the References list and also include a citation and full reference for the retraction notice.

Response: Thank you for this remark. We reviewed our paper carefully and checked all the references.

We did not change the reference list, except for the addition of a DOI in reference 15, (linking to a correction of supplementary materials).

Comments from Reviewer 2:

The revised version of the manuscript is improved and all my comments have been addressed. Thank you!

Response: Thank you very much for taking the time to review our manuscript, all the comments received in the reviewing process have helped us a lot.

Comments from Reviewer 3:

The manuscript is significantly improved after revision. However, I have two other comments to further improve it:

1/ The Introduction lacks of the objectives of the study (that have been added in the abstract only). Please add them.

Response: Thank you for this remark, we added the objectives of the study at the end of the Introduction section, at lines 54-59:

"The objectives of the study presented in this article are 1) to describe the characteristics of elderly multimorbid patients in the studied hospital using diagnoses information, multimorbidity and frailty scores, and 2) to assess the ability of machine learning models to predict rehospitalization within 30 and 365 days and patients' length of stay, using diagnoses information and two aggregative scores : the hospital frailty risk score and the Calderon-Larrañaga score."

2/ The Conclusion section is more than one page long, reporting also the results of the study including a discussion. I suggest to shortened them (moving the discussion of the results in the Discussion), reporting in this section only the conclusions and future perspectives/clinical application.

Response: The discussion and conclusion sections have been modified to improve the coherence of the paper, on pages 14-17. Comments on the results were moved to the discussion section as suggested, and we modified the structure of this section.

Thank you once again for your comments which have allowed us to considerably improve our paper.

Dr Jules Le Lay
