# Supplementary material for: Prediction of hospital readmission of multimorbid patients using machine learning models
Source: PLoS One. 2022 Dec 22;17(12):e0279433. doi: 10.1371/journal.pone.0279433 (PMC9779015; doi:10.1371/journal.pone.0279433)
Supplement: Attachment — Submitted filename: Response to reviewers.docx [file pone.0279433.s001.docx]

Jules Le Lay

École des Mines de Saint-Étienne

158 cours Fauriel,

42100 Saint-Étienne

Dear Editors of the PLOS ONE journal,

Thanks for the opportunity to submit a revised version of our manuscript entitled « Prediction of Hospital Readmission of Multimorbid Patients Using Machine Learning Models» by Le Lay, Alfonso-Lizarazo, Augusto, Bongue, Masmoudi, Xie, Gramont, and Célarier. We appreciate the efforts you and the rewiewers made to comment our manuscript, which have allowed us to improve the article.

Here under is a thorough response to the editors’ and reviewers’ comments :

**Editors:**

1) Please ensure that your manuscript meets PLOS ONE's style requirements, including those for file naming. The PLOS ONE style templates can be found at :

https://journals.plos.org/plosone/s/file?id=wjVg/PLOSOne_formatting_sample_main_body.pdf and

https://journals.plos.org/plosone/s/file?id=ba62/PLOSOne_formatting_sample_title_authors_affiliations.pdf

**R/ We added elements and renamed the files to meet the requirements. Please let us know if we missed any of the points listed.**

2) We suggest you thoroughly copyedit your manuscript for language usage, spelling, and grammar. If you do not know anyone who can help you do this, you may wish to consider employing a professional scientific editing service

**R/ The initial manuscript was edited for language, spelling and grammar by AJE editing services. We’ll attach the editing certificate to the resubmission. The modifications in this version concern mostly the figures and tables, and only a few sentences. Please let us know if another editing is required.**

3) Please ensure you include in the Methods section of your manuscript all information regarding the data access authorization you have provided in the Ethics Statement section of the online submission form, including information regarding the exception for patient consent granted by the authority who approved the data access.

**R/ We added in the Methods section the information regarding data access, this study was approved by the French national commission on informatics and liberty (CNIL) for the access to data under authorization number 919300. (This information was added to the revised paper and was already present on the online submission form).**

4) We note that you have indicated that data from this study are available upon request. PLOS only allows data to be available upon request if there are legal or ethical restrictions on sharing data publicly.

**R/ Indeed, the data used for the experiments presented in the paper are anonymized patients data extracted from medical records of the University hospital of Saint-Étienne. We were given access to this database by the 'Commission Nationale Informatique et Libertés' under authorization number 919300. The authors do not have the right to share this information publicly.**

**Reviewer 1:**

5) Page 9, line 31: the quotation mark before "an admissione" seems "inverted";

**R/ Indeed, I did not use the right quotation mark, this was corrected in the revised version of the paper.**

6) Materials and methods: in the "epidemiological description" section some results are described. I would suggest to adapt and move this part to the "Results" section.

**R/ We moved this paragraph at the beginning of the “Results” section as suggested.**

**Reviewer 2:**

7) I think that, for the models listed in tab 4, a figure showing the ROC curves may be beneficial for the readers as it would allow to understand if cut-offs other than predict probability = 0.5 are viable and what is their performance. This may be particularly interesting for the potential “clinical” application of these models where, according to one’s aim, a trade-off between sensitivity and specificity may be acceptable.

**R/ The ROC curves have been built for those models and have been included in the revised paper.**

8) I think that for the models listed in tab 4, reliability plots should be shown. This would allow the readers to better understand the reliability of the proposed models. It may also help the Authors to understand if any problem with calibration arises. RF models, for example, may need some kind of recalibration, and this issue may be particularly important given that the models were trained on “artificial data” with solved class imbalance and applied to “real” testing data. I’d suggest the Authors to try and see if Platt scaling or isotonic regression are viable ways to improve the calibration of the models (if any issue arise).

**R/ Calibration curves were plotted for those models and have been included in the revised paper, in the appendix.**

9) In general, models seem to be prone to overfitting (as expected). I would suggest to try and run the models including only a subsample of available variables (based on the importance of each variable, obtained after the fitting of complete RF models – for example best 5%, best 25%, best 50%). This may have several advantages: it may reduce the overfitting, may reduce the calculation time, and may have sense from a clinical point of view. Indeed, it has been proven that some chronic conditions (or group of conditions) are strongly linked to the risk of hospitalization (heart failure, COPD, dementia for example), whereas other are not (osteoarthritis for example). These latter conditions may even introduce some form of statistical noise in the models.

**R/ We think that this is an interesting improvement opportunity. However, unfortunately, we did not have enough time to develop the experimentation of this approach.**

10) Please, add 95% CI to the performance metrics listed in the paper (accuracy, F1, AUCs) to allow a better comparison between models.

**R/ We implemented a bootstrap method to calculate the 95% CI for the metrics used in the classification metrics. This information was included in the revised paper.**

11) I’d like to know what is the performance (Accuracy, F1, AUC) of the simple count of chronic conditions according to the Calderon-Larrañaga’s list of chronic diseases categories (for example, used in a simple logistic regression). This also goes for age used as single predictor. From an application point of view, it is important to know what the actual benefit of the implementation of ML models in comparison with much more simpler measures is.

**R/** **Our work focuses on the use of classical machine learning methods on medico-economic datasets enriched with medical tools used in the description of multimorbid patients. In this way, although the comparison with other methods is not part of our objectives, we carried out an experiment using logistic regression for hospital readmission within 30 days with random undersampling using all diagnosis: and obtained the following results (which have not been included in the article)**

|  | Accuracy | F1-score | ROC-AUC SCORE |
| --- | --- | --- | --- |
| Balanced Training set | 0.686 [0.670 – 0.704] | 0.680 [0.661 – 0.698] | 0.686 [0.670 – 0.703] |
| Training set | 0.636 [0.626 – 0.645] | 0.371 [0.636 – 0.663] | 0.649 [0.636 – 0.663] |
| Testing set | 0.600 [0.585 – 0.617] | 0.291 [0.262 – 0.322] | 0.577 [0.550 – 0.603] |

**Based on these results, the logistic regression with random undersampling using all diagnosis provides lower performances with respect to the method presented in the article.**

12) For the prediction of LoS, the performance shown by the models is not optimal (as highlighted by the Authors). Here, I would suggest, as sensitivity analyses, to either exclude or aggregate those participants with very long LoS (for example, outliers with LoS > Q75 + 2*IQR). From a clinical point of view, these very long LoS are likely to be associated with hospitalizations characterized by adverse events and complications (delirium, nosocomial infections and so on).

**R/ Although in our dataset a very small number of patients have a very long LoS compared to the average patient, we decided not to exclude or aggregate records. Indeed, we believe it is important to include these records in the training of machine learning algorithms to better identify characteristic patterns of prolonged stays.**

13) The Calderon-Larrañaga list of chronic conditions has been created using data from a population-based study enrolling only persons aged 60+. Has the HFRS been validated in younger persons? If not, it would be preferable to exclude patients younger than 60 from the analysis as these persons are more likely to be affected by a few chronic conditions with a strong impact on health, whereas those older are more likely to accumulate an elevated number chronic conditions.

**R/ We have taken this remark into account and for this revised paper we have carried out the experimentation excluding patients younger than 60 and reported them in the revised paper. The results, in comparison to those taking into account the entire population (including patients younger than 60), are slightly lower or slightly higher depending on the indicator under analysis. For example, the mean absolute error and mean square error were improved for LoS prediction. Accuracy and F1-score were approximately the same for 365 days hospital readmission, with a slight improvement in the best-case scenario (using all diagnosis and random forest). Accuracy, F1-score and ROC-AUC for 30-days readmission have slightly decreased.**

14) In some Countries, the diagnoses reported on the discharge letter from hospitals are used to obtain some kind of public (re)fundings. This is likely to introduce a bias in the reported diagnoses: those diagnoses that lead to higher (re)fundings are more likely to be reported. Is this the case also for the setting used by the Authors?

**R/ The anonymized patient data used in our study is extracted from the hospital electronic records. The methodology used in this study does not allow us to identify the existence of this kind of bias or to quantify it if it exists.**

15) It would be interesting to look at the predictive performance of the models in different age strata of the population. It is likely that among very old individuals with multiple chronic conditions (and likely affected by frailty and disability), a general tendency toward non-hospitalization and at-home (or nursing home) management is present. This may lead to a paradox where younger persons with multimorbidity are likely to be (re)hospitalized, whereas older persons are likely to be (re)hospitalized only when they are “healthier”.

**R/ As mentioned in the response to comment 13, in the revised paper we have presented the results excluding patients younger than 60, for this reason we have decided not to perform an analysis by age strata.**

**Reviewer 3:**

1) The Introduction should be improved: the authors should explain the rationale of using machine learning to predict hospitalizations and length of stay, expand what it is known on this topic and what this study will add to the literature. Furthermore, Introduction should include the objectives of the study, that are lacking. Reading the Abstract, it is not clear which is the objective of the study. To compare different machine learning predicting models.

**R/ The introduction section has been modified including these elements.**

**The objective of this study is the prediction of rehospitalization within 30 and 365 days and length of stay of multimorbid patients using machine learning models in which multimorbidity is measured using all diagnoses information (ICD-10 codes) and two aggregated multimorbidity and frailty scores.**

2) Epidemiological description included results such as the total number of the participants and their mean age. This information should be moved in the Results.

**R/ We moved this section at the beginning of the “Results” section.**

3) There is no information about ethic committee approval.

**R/ The study was approved by the French national commission on informatics and liberty (CNIL) for the access to data under authorization number 919300. This information was added to the revised paper.**

4)	Why using three different methods to classify multimorbidity?

**R/ Since our study focuses on multimorbidity patients, it is important for us to consider different approaches reported in the literature to measure multimorbidity and evaluate which of them allow a better prediction of re-hospitalization for this type of patients.**

5) Which were the variables included in the learning experiments?

**R/ We have included in the revised paper (section materials and methods) an explicit list of the variables used in the models.**

6) General characteristics of the study sample should be reported. Otherwise, it is very difficult to interpret the results.

**R/ We reported in the paper the general characteristics of the dataset in section “Results/Epidemiological description” the epidemiological features.**

7) Prediction of readmission within 30 days: I think that could be more appropriate to describe resampling, the metrics used, etc in the Methods section and to report in this subsection (and in the Results section in general) only the results.

**R/** **This comment has been considered in the revised paper.**

8) The first paragraph of the discussion should report a summary of the main results of the study.

**R/** **This comment has been considered in the revised paper.**

9) It seems that the conclusions simply discuss the results of the study (thus, most part of this section should be moved to the Discussion, comparing them with the available literature).

**R/ This comment has been considered in the revised paper.**

10) I suggest to report all the limitations of the study in a single paragraph and not throughout the Discussion.

**R/ This comment has been considered in the revised paper.**

11) The conclusion should briefly summarize the conclusions of the study, potential clinical implications and future perspectives.

**R/ This comment has been considered in the revised paper.**

12) In the conclusions the authors stated that they reported the general characteristics of the multimorbidity population, but I can’t find this information.

**R/ The general characteristics of the multimorbidity population studied are described in the “Results” section, in the “Epidemiological description” subsection.**

We would like to thank all reviewers for their detailed comments, we believe the modifications made to the experimental design and reporting of the results improves the quality of the paper.

We look forward to hearing from you regarding our submission and to respond to any additional questions or comments you may have.

Jules Le Lay, Edgar Alfonso-Lizarazo, Vincent Augusto1, Bienvenu Bongue, Malek Masmoudi, Xiaolan Xie, Baptiste Gramont and Thomas Célarier.
